# Pharmacognostic Characterization of *Abutilon theophrasti* Medic. Growing in Kazakhstan

**DOI:** 10.3390/plants15142110

**Published:** 2026-07-08

**Authors:** Akdidar Yegizbayeva, Nurgali Rakhymbayev, Kairat Zhakipbekov, Galiya Ibadullayeva, Elmira Serikbayeva, Arailym Yegizbayeva, Arailym Mukanova, Nazgul Makasheva, Nurdaulet Zhumabayev, Bassymbek Dilbarkhanov

**Affiliations:** 1 School of Pharmacy, Asfendiyarov Kazakh National Medical University, Tole bi 94, Almaty 050012, Kazakhstan; egizbayeva.a@kaznmu.kz (A.Y.); serikbaeva.e@kaznmu.kz (E.S.); mukanova.a@kaznmu.kz (A.M.); makasheva.n@kaznmu.kz (N.M.); 2 Department of Anatomy with Courses, Kazakh-Russian Medical University, Abylai khan 51/53, Almaty 050000, Kazakhstan; arailymegizbaeva@mail.ru; 3 Center for Life and Health Sciences, National Academy of Sciences of Kazakhstan Under the President of the Republic of Kazakhstan, Shevchenko 28, Almaty 050010, Kazakhstan; nurdaulet_phd@mail.ru; 4 Department of Therapeutic and Surgical Dentistry, Astana Medical University, Beibitshilik 49A, Astana 010000, Kazakhstan; bassimbek@gmail.com

**Keywords:** *Abutilon theophrasti* Medic., macroscopical analysis, microscopical analysis, histochemical analysis, pharmacognostic characteristics

## Abstract

*Abutilon theophrasti* Medic. is a widely distributed species belonging to the Malvaceae family. It represents a potential source of bioactive compounds with antioxidant, anti-inflammatory, and antimicrobial properties. Despite the species’ pharmacological significance and its widespread distribution, information on the diagnostic pharmacognostic characteristics of *A. theophrasti* Medic. remains limited, and no such studies have previously been conducted on raw materials growing in Kazakhstan. The objective of this study was to ascertain the diagnostically significant morphological, anatomical, and histochemical characteristics of *A. theophrasti* Medic. in order to facilitate the identification and pharmacognostic standardization of medicinal plant raw materials. The subjects of the study were the plant’s aerial and underground organs, including leaves, stems, petioles, inflorescences, flowers, fruits, and roots. A comprehensive analysis of the raw material was conducted using a variety of analytical methods, including macroscopic, microscopic, and histochemical approaches. The study’s findings enabled the identification of characteristic features of the plant’s aerial and underground organs through macroscopic analysis. Microscopic examination revealed diagnostically significant characteristics of the species *A. theophrasti* Medic., including an anomocytic stomatal apparatus, numerous simple and stellate trichomes, calcium oxalate druses, and characteristic anatomical features of the leaf, stem, sepals, and root. Taken together, these characteristics form a diagnostic complex that can be used to identify this species. Histochemical analysis revealed the localization of phenolic compounds, flavonoids, and trace amounts of essential oil in the tissues of the leaf petiole and root. However, alkaloids, starch, and sesquiterpene lactones were not detected. The identified morphological, anatomical, and histochemical data serve to supplement the information on the diagnostically significant characteristics of the species, carrying practical importance for the quality assessment of plant raw materials.

## 1. Introduction

The study of medicinal plants remains one of the key areas of modern pharmacognosy and pharmaceutical science [1,2]. The flora of Kazakhstan is characterized by a high level of biodiversity and includes more than 5700 species of higher vascular plants, of which at least 1406 species are medicinal plants, accounting for about a quarter of the country’s vascular plant flora [3]. Despite significant resources of medicinal plant raw materials, many species remain insufficiently studied from a pharmacognostic perspective. In this regard, special attention is given to understudied plant species of interest as potential sources of medicinal plant raw materials.

**Figure 1:**
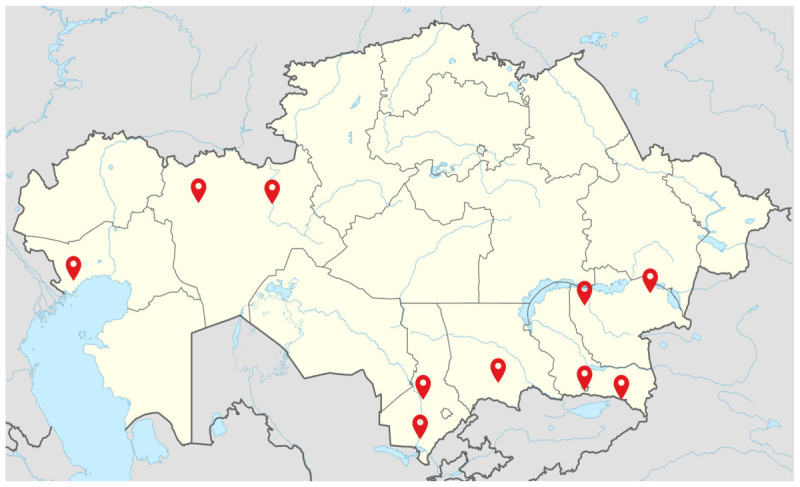
Geographic distribution and sampling sites of *A. theophrasti* Medic. in Kazakhstan (adapted from NordNordWest/Wikimedia Commons, CC BY-SA 3.0 DE).

Of particular interest for pharmacognostic and phytochemical studies are species of the genus *Abutilon*, which is one of the largest in the Malvaceae family and includes 150 to 200 species of herbaceous plants, shrubs, and small trees [4,5,6]. Species of this genus are distributed primarily in the tropical and subtropical regions of the Americas, Africa, Asia, Australia, India, and some areas of Southern Europe, where they grow under various ecological conditions, indicating their high adaptability and ecological plasticity [6,7,8,9,10]. According to floristic data, the genus *Abutilon* is represented in the flora of Kazakhstan by only one species, *A. theophrasti* Medic., which is distributed primarily in the southern and western regions of the country [11] (Figure 1).

Although information on the chemical composition and pharmacological activity of this species is available, the pharmacognostic characteristics of the raw material from Kazakhstani populations have not yet been studied. Given that *A. theophrasti* Medic. is the sole representative of the genus in the flora of Kazakhstan, the study of its morphological, anatomical, and histochemical characteristics is important for establishing the diagnostic criteria necessary for the identification, authentication, and subsequent pharmacognostic standardization of medicinal plant raw materials.

Members of the *Abutilon* genus (Malvaceae) are characterized by a wide range of biological activities, including antioxidant [12,13], anti-inflammatory [14,15,16], antimicrobial [14,17,18], antidiabetic [19,20], antitumor [21,22], immunomodulatory [23], diuretic [24], antiulcer [25,26], antiasthmatic [27], antitubercular [28], anticonvulsant [29], antiparasitic [30], and wound-healing effects [14,31]. Due to these properties, various plant parts have long been used in traditional medicine across different countries: roots and bark serve as diuretics and astringents; leaves and flowers are applied to treat inflammatory conditions and skin lesions; and decoctions or infusions from seeds and fruits are utilized for dysentery, dyspepsia, diabetes mellitus, and gastric disorders [32]. This diverse therapeutic potential is primarily attributed to the presence of flavonoids, saponins, phenolic acids, and other biologically active metabolites [33]. Among the most prominent representatives of the genus is *A. theophrasti* Medic., which has a rich history of traditional use specifically for treating wounds, inflammatory processes, skin diseases, and gastrointestinal disorders [4]. According to phytochemical studies, more than 100 biologically active compounds have been identified in plants of the genus *Abutilon*, including *A. theophrasti* Medic. This species contains flavonoids, phenolic acids, alkaloids, coumarins, triterpenoids, sterols, saponins, glycosides, and tannins, as well as components of fatty and essential oils [34,35,36].

Despite the interest in plants of the genus *Abutilon*, the morphological, anatomical, histochemical, and pharmacognostic characteristics of *A. theophrasti* Medic., which grows in Kazakhstan, have not been previously studied. The introduction of new types of medicinal plant raw materials into pharmaceutical practice requires their pharmacognostic standardization and the development of diagnostic criteria for authenticity. Macroscopic and microscopic analyses remain among the primary methods for identifying medicinal plants [37,38,39].

Macroscopic and microscopic analysis of medicinal plant raw materials allows for the identification of diagnostically significant characteristics necessary for the identification and standardization of raw materials [40,41]. Histochemical studies make it possible to determine the localization of biologically active compounds in the tissues of individual plant organs [41,42]. In this regard, the study of the morphological, anatomical, and histochemical characteristics of *A. theophrasti* Medic., which grows in Kazakhstan, is of interest for the identification and pharmacognostic standardization of this species.

The objective of the present study was to ascertain the diagnostically significant morphological, anatomical, and histochemical characteristics of *A. theophrasti* Medic. growing in Kazakhstan for the identification, authentication, and pharmacognostic standardization of medicinal plant material. The study included the plant’s aerial and underground parts, including leaves, stems, petioles, inflorescences, flowers, fruits, and roots.

## 2. Results

### 2.1. External Morphology of A. theophrasti Medic. Plant Parts

**Figure 2:**
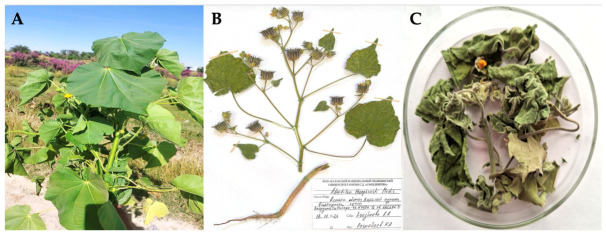
*A. theophrasti* Medic. growing in natural conditions (**A**), herbarium specimen of the plant (**B**), and ground raw material of *A. theophrasti* Medic. (**C**).

*A. theophrasti* Medic. is an annual plant, growing up to 100–120 cm in height (Figure 2A). The stem is straight, cylindrical, and simple; it may branch in the upper part with short flowering branches, which are pubescent with short trichomes, and in the upper part with dense glandular trichomes (Table 1). Leaves have long petioles; the leaf blades are broadly ovate with a cordate base, up to 15 cm long, with a pointed apex and shallowly crenate margins and are velvety on both sides due to dense pubescence (Table 1). Flowers are arranged in racemose or panicle inflorescences (Figure 2C, Table 1). Flowers have a double perianth; the sepals are fused up to halfway, with the terminal lobes ovate and ending in a short point. The corolla is 8 to 15 mm long, light yellow, with fused petals (Table 1). The fruit is a capsule, star-shaped at the top (Figure 2B, Table 1).

The results of the morphological analysis of *A. theophrasti* Medic., presented in Table 1, show that the plants possess characteristic macroscopic structural features.

### 2.2. Microscopic Description of A. theophrasti Medic.

#### 2.2.1. Anatomical Characteristics of the Leaf

**Figure 3:**
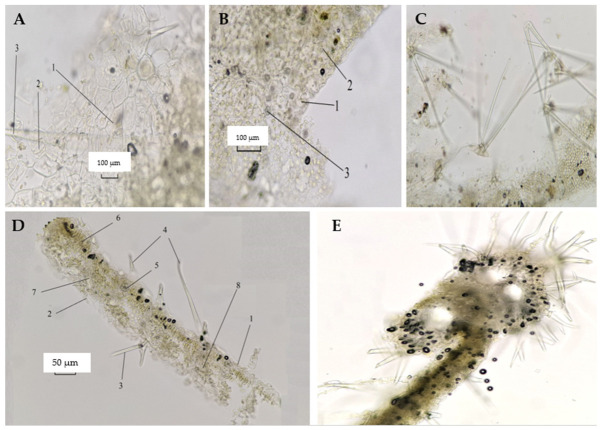
Leaf micromorphology of *A. theophrasti* Medic.: (**A**–**C**) Surface preparation: (**A**) upper epidermis: 1—epidermal cells, 2—stoma, 3—trichome; (**B**) lower epidermis: 1—epidermal cells, 2—stoma, 3—calcium oxalate druse; (**C**) simple and stellate trichomes along the leaf veins. (**D**,**E**) Cross-section: (**D**) lateral section of the leaf blade: 1—upper epidermis, 2—lower epidermis, 3—stellate trichome, 4—simple trichomes, 5—calcium oxalate druse, 6—columnar mesophyll, 7—spongy mesophyll, 8—lateral vascular bundle; (**E**) fragment in the region of the midrib.

A surface preparation of the upper and lower surfaces of a leaf of *A. theophrasti* Medic. (Figure 3A,B) shows epidermal cells that are oval or polygonal in shape, with thin walls. Stomata of the anomocytic type are distributed across the surface, with a greater number located on the lower surface (hypostomatic type).

Trichomes are numerous, located on both sides of the leaf; they are simple, unicellular, and multicellular and are clearly visible, particularly along the leaf veins (Figure 3C). Rounded druses of calcium oxalate are visible on the lower surface.

In cross-section, the leaf is light-colored and of the dorsoventral type; the mesophyll is differentiated into columnar and spongy tissues (Figure 3D). The cells of the upper and lower epidermis form a single layer and are rounded in shape. The surface is covered with numerous simple and stellate trichomes, well-defined along the veins (Figure 3E). The columnar mesophyll lies beneath the upper surface and is arranged in a single row. The spongy mesophyll consists of 2–3 layers and lies beneath the lower epidermis. Rare, rounded calcium oxalate druses are visible in the mesophyll. The vascular bundles are rounded, collateral, of the closed type, and surrounded by sclerenchyma strands.

#### 2.2.2. Anatomical Characteristics of the Flower

**Figure 4:**
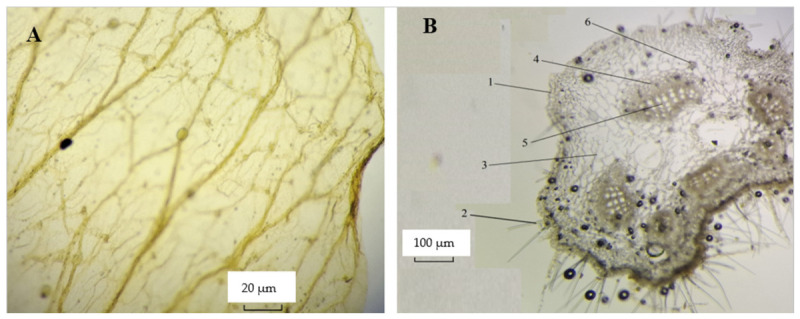
Flower micromorphology of *A. theophrasti* Medic.: (**A**) corolla surface preparation; (**B**) calyx lobe cross-section: 1—epidermis, 2—trichome, 3—mesophyll, 4—phloem, 5—xylem, 6—calcium oxalate druse.

The surface of the flower’s corolla consists of a layer of epidermal tissue that is rounded in shape, with cells tightly packed together. Pollen grains may be present on the surface (Figure 4A).

The calyx lobes are rounded–triangular in cross-section (Figure 4B). Externally, they are covered by a single-layered epidermis composed of rounded cells with thickened outer walls. The mesophyll is undifferentiated and consists of oval parenchyma cells. Four vascular bundles, oval or broadly ovate in shape, sometimes slightly curved, collateral, and of the closed type, are located in the mesophyll. Rare druses of calcium oxalate are also noted in the mesophyll.

#### 2.2.3. Anatomical Characteristics of the Stem and Root

**Figure 5:**
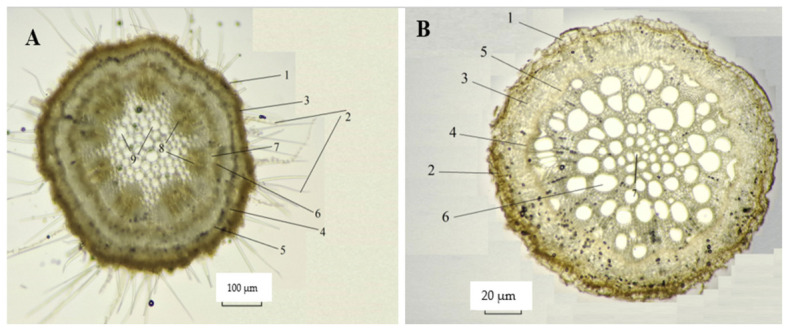
Transverse section of stem (**A**) and root (**B**) of *A. theophrasti* Medic.: (**A**) 1—epidermis, 2—trichomes, 3—chlorenchyma, 4—cortical parenchyma, 5—endodermis, 6—sclerenchyma, 7—phloem, 8—xylem, 9—medullary parenchyma; (**B**) 1—rhizodermis, 2—periderm, 3, 4—cortical parenchyma, 5—phloem, 6—secondary xylem vessels, 7—primary xylem vessels.

The stem of *A. theophrasti* Medic. is rounded in cross-section (Figure 5A). The surface is covered by a single-layered epidermis composed of oval cells. Numerous simple unicellular and multicellular trichomes extend from the epidermis. Beneath the epidermis lie 2–4 layers of chlorenchyma, followed by a multilayered cortical parenchyma, which accounts for up to 10% of the total stem volume. The primary cortex is separated from the vascular zone by a single-layered endodermis. Its cells are oval, small, and clearly visible in a micro-preparation. The vascular bundles are broadly ovoid in shape, collateral (of the open type, with fascicular cambium present), with the phloem oriented outward and the xylem inward. Above each vascular bundle lie areas of sclerenchyma, forming broad “caps.” In the central part, a broad parenchyma core is present, consisting of rounded, thin-walled cells.

The root has a rounded cross-section (Figure 5B). Remnants of the outer dead tissues (remnants of primary cortex and exfoliated rhizodermis) can be seen on the surface, while the main part is covered by the periderm, which is brown or dark brown in color. Beneath the periderm lie areas of primary and secondary parenchyma. The vascular system exhibits a secondary structure, consisting of radially diverging phloem rays and secondary xylem vessels, reinforced by individual sclerenchyma cells. Areas of primary xylem are preserved in the central part.

### 2.3. Histochemical Characterization of A. theophrasti Medic.

The study revealed characteristic staining patterns in various cell types, resulting from the interaction of the reagents with the metabolites being detected [43,44,45,46,47]. The results of the histochemical analysis for the identification of specific groups of metabolites in the petiole and root of the plant under study are presented in Table 2.

**Figure 6:**
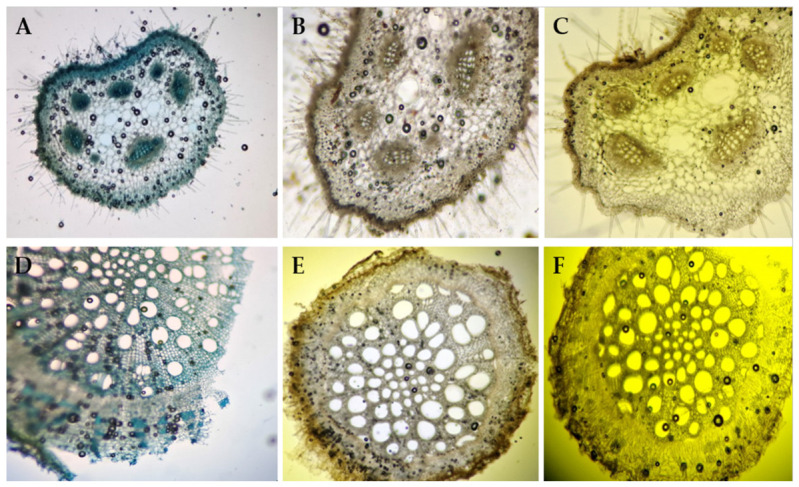
Histochemical characterization of vegetative organs of *A. theophrasti* Medic.: (**A**–**C**) Leaf petiole: (**A**) cross-section of the leaf petiole stained with methylene blue; (**B**) cross-section of the leaf petiole stained with a 1% alcohol solution of FeCl_3_; (**C**) cross-section of the leaf petiole stained with a 10% alcohol solution of K_2_Cr_2_O_7_. (**D**–**F**) Root: (**D**) cross-section of the root stained with methylene blue; (**E**) cross-section of the root stained with a 1% alcohol solution of FeCl_3_; (**F**) cross-section of the root stained with a 10% alcohol solution of K_2_Cr_2_O_7_.

The results of a histochemical study of the leaf petiole of *A. theophrasti* Medic. revealed the presence of phenolic compounds, flavonoids, and traces of essential oil (Figure 6A–C). Characteristic staining of individual tissue areas was observed using methylene blue, a 1% alcohol solution of FeCl_3_, and a 10% alcohol solution of K_2_Cr_2_O_7._ Traces of essential oil were detected upon staining with methylene blue (Figure 6A). The reaction with a 1% alcohol solution of FeCl_3_ indicated the presence of flavonoids in the leaf petiole tissues (Figure 6B). The presence of phenolic compounds was determined using a 10% alcohol solution of K_2_Cr_2_O_7._ (Figure 6C). Similar studies were conducted on the root (Figure 6D–F) of *A. theophrasti* Medic.: as in the leaf petiole, an accumulation of phenolic compounds, essential oils, and flavonoids was observed; starch, sesquiterpene lactones, and alkaloids were not detected.

The results of histochemical reactions showed that traces of essential oil are localized in the tissues of the leaf petiole (Figure 6A–C) and the root (Figure 6D–F). Thus, in the root, characteristic staining was observed in the cortical parenchyma and periderm and in the leaf petiole in the mesophyll near the epidermis, trichomes, and vascular bundles. Staining with iron chloride revealed significant localization of flavonoids in the periderm and cortical parenchyma and, to a lesser extent, in the xylem tissues (Figure 6E). In the leaf petiole (Figure 6B), the epidermis and vascular bundles were stained, with a smaller amount in the mesophyll. Phenolic compounds in the root (Figure 6F) are localized in the periderm and sclerenchyma; in the leaf petiole (Figure 6C), they are found in the epidermis, mesophyll, and xylem. Sesquiterpene lactones, starch, and alkaloids were not detected using histochemical reactions.

## 3. Discussion

The morphological and anatomical characteristics identified in *A. theophrasti* Medic. are of significant interest from the perspective of pharmacognostic identification of plant raw materials. The most informative diagnostic characteristics were found to be the structure of the leaf epidermis, the type of stomatal apparatus, the features of the trichome complex, and the presence of calcium oxalate druses. Such microscopic characteristics are considered reliable diagnostic markers, as they remain intact after the raw material is ground and can be used to confirm its authenticity during microscopic analysis [48,49].

The identified anomocytic stomatal type, as well as the presence of simple multicellular and stellate trichomes, are generally consistent with the results of previous studies on *A. theophrasti* Medic. [48,50]. Studies on species of the genus *Abutilon* have shown that the morphology, type, and distribution of trichomes are among the most informative characteristics of taxonomic and pharmacognostic significance [49,51,52]. For *A. theophrasti* Medic., the presence of multicellular uniseriate trichomes is also noted, which corresponds to the results of the present study [49]. The presence of stellate trichomes is considered a characteristic feature of species of the genus *Abutilon* and the family Malvaceae [49,52]. Beyond their taxonomic and diagnostic value, these epidermal structures play a vital ecophysiological role. The dense indumentum formed by glandular and non-glandular trichomes is crucially important for herbivore defense and tolerance to environmental stress, thereby significantly contributing to the high ecological adaptability and hardiness of *A. theophrasti* [51]. The presence of calcium oxalate druses in the tissues of the leaf and calyx has additional diagnostic significance. Similar crystalline inclusions have previously been detected in various organs of *A. theophrasti* Medic. [48,50], indicating their stability within the species. Furthermore, in pharmacognostic studies of other members of the genus *Abutilon*, crystalline inclusions, epidermal features, trichomes, and the structure of vascular tissues are also considered important characteristics for the identification of plant material [51].

The identified features of the anatomical structure, including the dorsiventral type of mesophyll, the presence of enclosed collateral vascular bundles, and well-developed mechanical tissues, generally correspond to the data published for *A. theophrasti* Medic. [48,50], as well as to pharmacognostic studies of closely related species of the genus *Abutilon* [51,52]. The combination of identified characteristics confirms their diagnostic value and may serve as a basis for subsequent pharmacognostic standardization of the plant material of this species.

The results of histochemical analysis revealed the localization of phenolic compounds, flavonoids, and traces of essential oil in the root and petiole tissues of *A. theophrasti* Medic. The detection of phenolic compounds and flavonoids is consistent with data from phytochemical studies, according to which gallic, protocatechuic, caffeic, and ferulic acids, as well as catechin, rutin, quercetin, and other polyphenolic compounds, have been identified in various organs of *A. theophrasti* Medic. [34,35]. Their localization in the epidermis, mesophyll, cortical parenchyma, and vascular tissues indicates the accumulation of secondary metabolites in structures that provide plant defense and participate in substance transport.

Previous studies have demonstrated the potent antioxidant properties of proanthocyanidins [12], the anti-inflammatory activity of the flavonoid fraction [17], and the antimicrobial activity of phenolic extracts [18] in *A. theophrasti* Medic. The detection of flavonoids and phenolic compounds directly in the tissues of the studied organs confirms the results of phytochemical studies and allows us to link the established localization of these metabolites to the biological activity previously described for this species.

Despite the availability of published data on the morphology, anatomy, phytochemical composition, and pharmacological activity of *A. theophrasti* Medic. [48,51], information on the morphological, anatomical, and histochemical characteristics of plants growing in Kazakhstan was previously lacking. Furthermore, the available literature focuses primarily on the identification of biologically active compounds and the study of their pharmacological properties, whereas data on the tissue localization of secondary metabolites for this species are limited. The results obtained supplement existing knowledge on the diagnostically significant characteristics of *A. theophrasti* Medic. and expand the data necessary for its pharmacognostic evaluation. The histochemical analysis conducted allowed us to determine the localization of phenolic compounds, flavonoids, and traces of essential oil in the root and leaf petiole tissues, which complements previously published data on the plant’s chemical composition. The set of established macroscopic, microscopic, and histochemical characteristics can be used in the identification and standardization of plant raw materials of this species, as well as serve as a basis for further phytochemical and pharmacological studies.

## 4. Materials and Methods

### 4.1. Materials

The study examined both the aboveground and underground parts of *A. theophrasti* Medic., including the aboveground parts, namely leaves, stems, petioles, inflorescences, flowers, and fruits, and the underground parts: roots. This herbal material was collected in the village of Kyrgauyldy, Karasai District, Almaty Region, Republic of Kazakhstan (43.164894° N, 76.722646° E) during the flowering and fruiting period of this species (Figure 2). The species was identified at the Institute of Botany and Phytointroduction by Gulnara Sitpaeva on 18 October 2024, and a letter confirming the identification was issued (01-05/534). Herbarium specimens of the plant have been deposited in the collection of the Institute of Botany and Phytointroduction.

### 4.2. Macroscopic (Morphological) Analysis

Macroscopic analysis of *A. theophrasti* Medic. was performed visually and using a magnifying glass (^x^10) in accordance with the requirements of the State Pharmacopoeia of the Republic of Kazakhstan. Photography of the samples under study was performed using a “Biomed-4” microscope (BIOMED, Saint Petersburg, Russia) equipped with achromatic objectives and wide-field eyepieces. The obtained images were processed and edited using Altami Studio (v3.5.0, Altami Ltd., Saint Petersburg, Russia) and Paint 10.1 software. When studying morphological characteristics, the shape, size, color, surface texture, and degree of pubescence of the leaves, stems, petioles, inflorescences, flowers, fruits, and roots of the plant were taken into account.

### 4.3. Microscopic Analysis

Dried samples of *A. theophrasti* Medic. were first soaked in water and then softened in a mixture of 70% ethanol, glycerin, and distilled water in a 1:1:1 ratio (Strauss–Fleming mixture) [53,54]. Surface preparations and transverse sections of the organs under study were prepared manually using a razor blade. Microscopic preparations were examined using a “Biomed-4” microscope at magnifications of 16 × 4 and 16 × 10. Microphotographs were obtained using Altami Studio software, and subsequent image processing was performed in Paint 10.0. In describing the anatomical structure, the principles outlined in relevant scientific works were applied [37,38,39].

### 4.4. Histochemical Analysis

Dried plant material of *A. theophrasti* Medic. was rehydrated and fixed in a mixture of 70% ethanol, glycerin, and distilled water in a 1:1:1 ratio (Strauss–Fleming mixture) [43,44,45,46,47]. Histochemical analysis was performed on cross-sections of leaf petioles and roots. The following reagents were used during histochemical analysis:

- Methylene blue for the detection of essential oils;
- 1% alcoholic solution of FeCl_3_ for the identification of flavonoids;
- 10% alcoholic solution of K_2_Cr_2_O_7_ for the detection of phenolic compounds;
- Lugol’s solution for the identification of starch;
- Vanillin solution in concentrated H_2_SO_4_ for the detection of sesquiterpene lactones;
- Dragendorff’s reagent for the identification of alkaloids.

Changes in the color of individual tissues served as an indication of the localization of the corresponding groups of metabolites in the tissues of *A. theophrasti* Medic. Photographs of cross-sections of leaf petioles and roots were taken using a “Biomed-4” microscope with 10^x^ and 20^x^ eyepieces and 4^x^, 10^x^, 20^x^, and 40^x^ objectives. The resulting images were edited in Paint 10.1.

## 5. Conclusions

In this study, a comprehensive macroscopic, microscopic, and histochemical analysis of the aerial and underground organs of *A. theophrasti* Medic. growing in Kazakhstan was performed for the first time. The morphological, anatomical, and histochemical data obtained expand the existing pharmacognostic knowledge of *A. theophrasti* Medic. and supplement scientific data on the regional populations of this species growing in Kazakhstan.

The identified diagnostic features can serve as reliable criteria for the identification, authentication, quality assessment, and pharmacognostic standardization of the plant raw material. Furthermore, the results of this study can be utilized as a scientific basis for the development and improvement of national pharmacopeial documentation and quality monographs for medicinal plant materials.

Further research should focus on a comprehensive quantitative phytochemical study of the species, the identification and characterization of its main biologically active compounds, and an assessment of its pharmacological properties. The findings obtained will contribute to the development of standardization parameters and a more complete evaluation of the pharmacological potential of *A. theophrasti* Medic.

## Figures and Tables

**Table 1:** Morphological characteristics of the aerial and underground parts of *A. theophrasti* Medic.

Plant Organ		Description
Stem	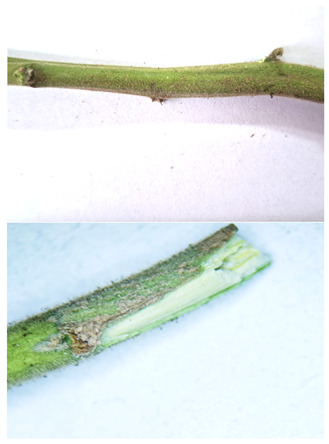	The stem is rounded, with simple whitish trichomes; the leaves are alternate; the fracture is white or beige.
Upper leaf surface	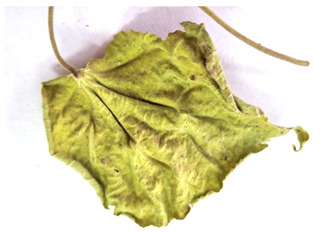	Palmate venation, simple white trichomes, green coloration.
Lower leaf surface	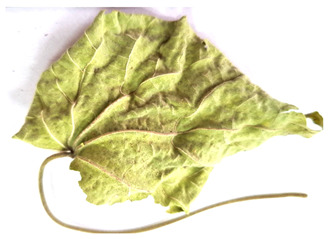	Prominent veins, densely covered with white trichomes, whitish-green in color.
Leaf petiole	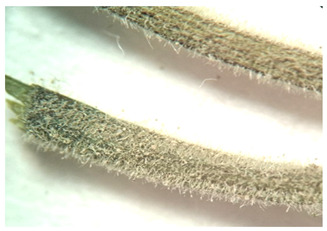	Cylindrical petioles with simple and glandular trichomes.
Inflorescence	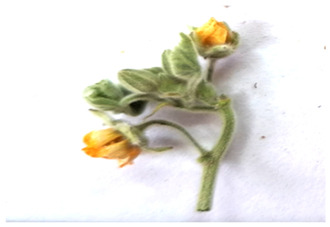	Terminal short racemes or panicles.
Flower	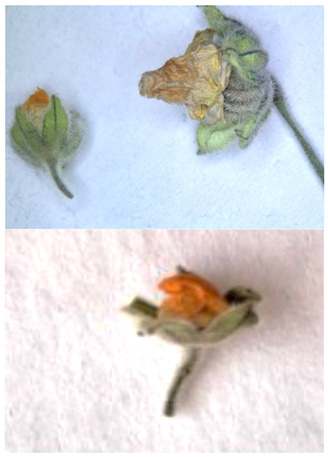	A hermaphroditic flower with a broad calyx and a yellow corolla that extends beyond the calyx.
Calyx	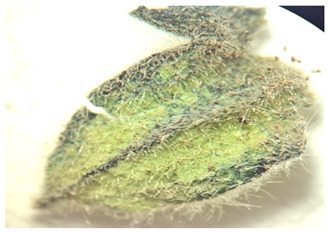	The sepals are ovate with pointed tips and densely covered with simple and glandular trichomes; they are light green in color.
Corolla	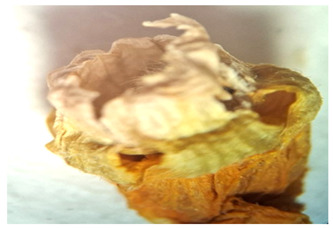	The corolla is yellow, smooth, and slightly ribbed.
Fruit	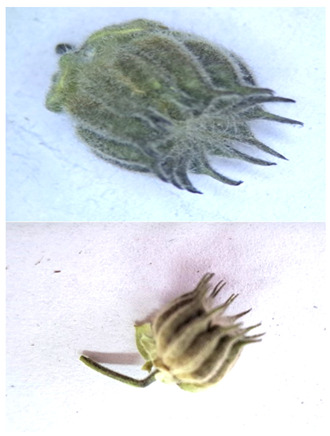	The fruit is a light green capsule featuring a star-shaped apex and covered with simple and stellate trichomes.
Root	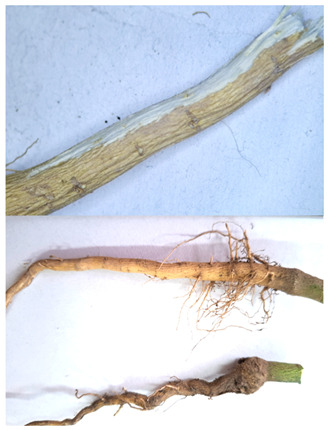	Taproot, sparsely branched, with a fibrous structure; bark grayish-brown, white when cut crosswise.

**Table 2:** Histochemical reactions in transverse sections of the leaf petiole and root of *A. theophrasti* Medic.

A Specific Group of Biologically Active Substances	Reagent	Root (Localization)	Leaf Petiole (Localization)
Essential oil	methylene blue reagent	Cortical parenchyma, periderm (phelloderm cells)	Mesophyll near the epidermis, trichomes, vascular bundles
Flavonoids	1% FeCl_3_ reagent	Periderm (phellem (cork) cells), cortical parenchyma, xylem tissues	Epidermis, vascular bundles, mesophyll
Phenolic compounds	10% K_2_Cr_2_O_7_ reagent	Periderm (phellem (cork) cells), sclerenchyma	Epidermis, mesophyll, xylem
Sesquiterpene lactones	vanillin solution in concentrated H_2_SO_4_ reagent	Not detected	Not detected
Starch	Lugol’s reagent	Not detected	Not detected
Alkaloids	Dragendorff’s reagent	Not detected	Not detected

## Data Availability

All tables and figures were created by the authors. All sources of information are properly cited. No copyright permissions are required. The original contributions presented in this study are included in the article. Further inquiries can be directed to the corresponding authors.
